# A Floating Bridge Disrupts Seaward Migration and Increases Mortality of Steelhead Smolts in Hood Canal, Washington State

**DOI:** 10.1371/journal.pone.0073427

**Published:** 2013-09-05

**Authors:** Megan Moore, Barry A. Berejikian, Eugene P. Tezak

**Affiliations:** Manchester Research Laboratory, Northwest Fisheries Science Center, National Oceanic and Atmospheric Administration Fisheries, Manchester, Washington, United States of America; University of Toronto, Canada

## Abstract

**Background:**

Habitat modifications resulting from human transportation and power-generation infrastructure (e.g., roads, dams, bridges) can impede movement and alter natural migration patterns of aquatic animal populations, which may negatively affect survival and population viability. Full or partial barriers are especially problematic for migratory species whose life histories hinge on habitat connectivity.

**Methodology/Principal Findings:**

The Hood Canal Bridge, a floating structure spanning the northern outlet of Hood Canal in Puget Sound, Washington, extends 3.6 meters underwater and forms a partial barrier for steelhead migrating from Hood Canal to the Pacific Ocean. We used acoustic telemetry to monitor migration behavior and mortality of steelhead smolts passing four receiver arrays and several single receivers within the Hood Canal, Puget Sound, and Strait of Juan de Fuca. Twenty-seven mortality events were detected within the vicinity of the Hood Canal Bridge, while only one mortality was recorded on the other 325 receivers deployed throughout the study area. Migrating steelhead smolts were detected at the Hood Canal Bridge array with greater frequency, on more receivers, and for longer durations than smolts migrating past three comparably configured arrays. Longer migration times and paths are likely to result in a higher density of smolts near the bridge in relation to other sites along the migration route, possibly inducing an aggregative predator response to steelhead smolts.

**Conclusions/Significance:**

This study provides strong evidence of substantial migration interference and increased mortality risk associated with the Hood Canal Bridge, and may partially explain low early marine survival rates observed in Hood Canal steelhead populations. Understanding where habitat modifications indirectly increase predation pressures on threatened populations helps inform potential approaches to mitigation.

## Introduction

Habitat modifications resulting from construction of transportation and power generation infrastructure (e.g. roads, dams, bridges) pose broad threats to aquatic animal populations because they can affect large areas, disrupt migration, and fundamentally influence behavior [1]–[3]. Migratory species are more likely to encounter and be affected by anthropogenic barriers than are species with smaller ranges. For example, North American sturgeon are well-adapted to large rivers and travel long distances in unobstructed rivers to exploit seasonally available resources [4]. However, dams without fish passage effectively prevent movement of sturgeon between historically available river segments, resulting in population isolation and habitat loss that have decreased genetic diversity [5] and increased risk of extinction [6].

Major anthropogenic structures can alter habitat for a wide range of taxa and influence ecological interactions among affected species. Pacific salmon and steelhead are highly migratory and provide clear examples of the effects of hydropower development on salmonid populations’ range [7], migratory behavior [1], [8], [9] and predator-prey interactions [10], [11]. Conversion of free-flowing rivers to impounded reservoirs alters the natural hydrograph, homogenizes habitat, increases water temperature and induces behavioral changes, including increased milling behavior near dam forebays [12] and slower migration rates [9]. Dams also create favorable conditions for piscine predators to congregate in slow-moving reservoir currents where they exploit migrating salmon and steelhead smolts [11], [13]. Adult salmon later congregate on their upriver migrations as they attempt to pass over Bonneville Dam, attracting increasing numbers of *Eumetopias jubatus* and *Zalophus californianus* (Stellar and California sea lions) that consume between 0.4–4.9% of the upriver salmon run each year [14]. Even partial barriers can affect migration; a floating bridge in Washington state was found to elicit milling behavior and slight migration delays in a portion of a migrating juvenile *O. tshwaytscha* (Chinook salmon) population in Lake Washington [15]. Whether habitat modifications introduce a complete or partial physical barrier or none at all, environmental factors are altered by human disturbance, causing behavioral changes that can inhibit natural movement.

**Figure 1:**
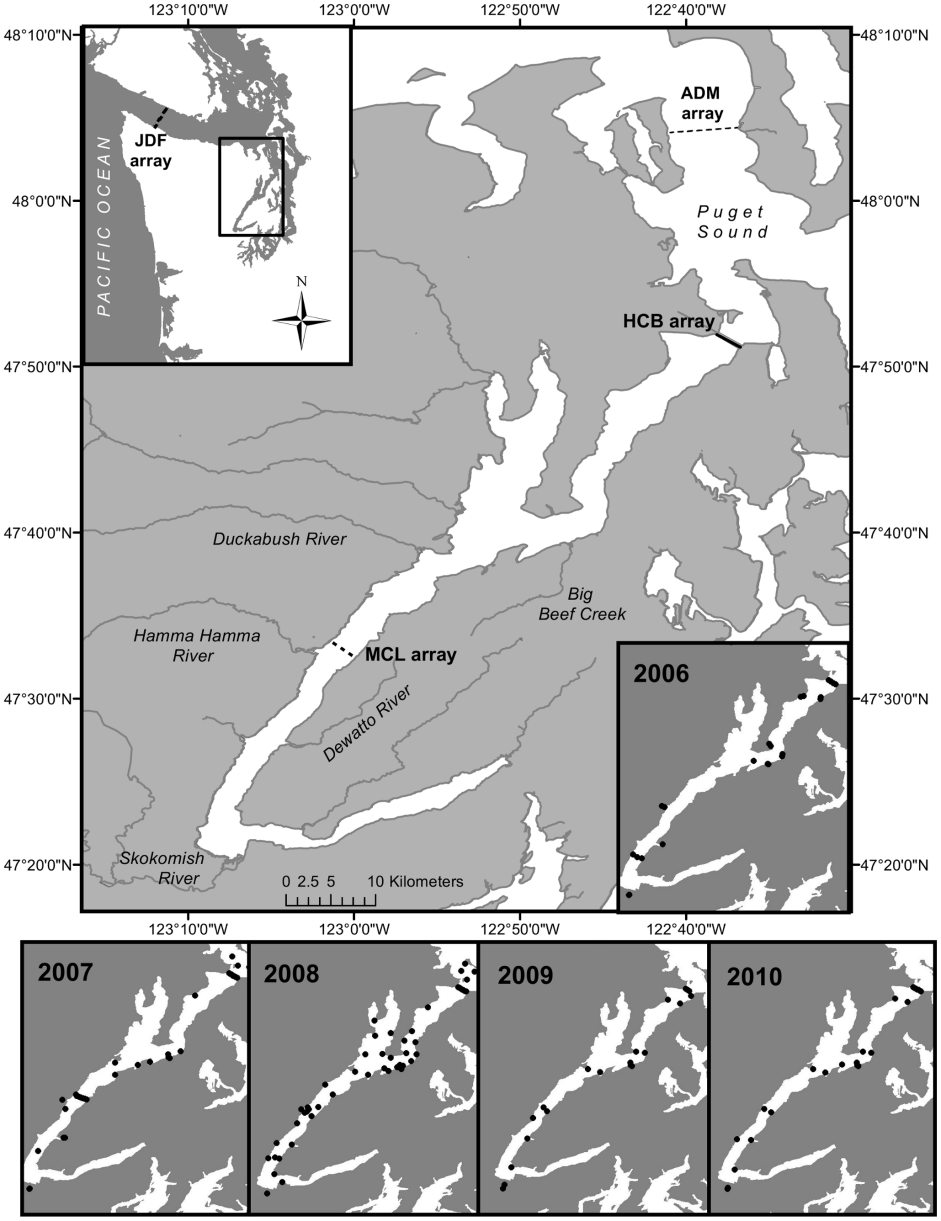
Map of study area. Map depicts the four receiver arrays: Hood Canal Bridge (HCB), Mid Canal (MCL), Admiralty Inlet (ADM), and Strait of Juan de Fuca (JDF). Lower insets show single receiver locations for each year.

Anadromous salmonids incur higher rates of mortality (deaths per unit time) upon seawater entry than during later phases of their marine migration [16], [17], which may largely determine survival to adulthood [18], [19]. Low early marine survival rates have been documented in threatened (ESA; FR 26722) populations of *Oncorhynchus mykiss* (steelhead) from Hood Canal [20], , central Puget Sound (Fred Goetz, unpublished data), and in nearby Georgia Basin steelhead populations [22], [23]. Steelhead migrating through the Salish Sea face a higher degree of human disturbance and habitat modification than do salmon and steelhead from coastal streams flowing directly to the Pacific Ocean. The Hood Canal is a fjord forming one of the four main basins of Puget Sound and has a floating bridge (built in 1961) to provide transportation from the Kitsap Peninsula to the Olympic Peninsula in Washington State. The bridge spans a 1.5 mile wide constriction near the northern outlet of Hood Canal (Fig. 1). Submerged concrete pontoons (3.6 m deep) support the bridge superstructure where vehicular traffic crosses the canal. The pontoons span roughly 95% of the width of Hood Canal at low tide, forming a substantial barrier to aquatic organisms travelling near the water’s surface. Recent telemetry studies on migrating juvenile steelhead in Hood Canal [20], [21] have reported behavioral anomalies associated with the Hood Canal Bridge that indicate migration disruption. Vertical distributions of migrating steelhead smolts have not been well documented, but juvenile salmonids are known to primarily inhabit the upper 12 meters of the marine water column [24], and are thus likely to encounter the submerged pontoons of the Hood Canal Bridge (HCB). In this paper we analyze data from five years of tagged steelhead outmigrations (2006–2010; [20], [21] to determine the probability that the Hood Canal Bridge impedes migration and contributes to extra mortality for steelhead smolts.

## Methods

Appropriate scientific collection permits were obtained from the Washington Department of Fish and Wildlife. The study plan and animal care procedures were approved by the NOAA Fisheries Northwest Fisheries Science Center. NOAA Fisheries also approved a Hatchery and Genetic Monitoring Plan for this study, which satisfies the criteria of the 4(d) rule under the Endangered Species Act. Fish were held for a minimal amount of time before tagging, anesthetized fully during surgery, and allowed to completely recover before release. No tagged smolt perished before release as a result of the surgeries performed in this study, and all appeared to be alert, behaving normally, and in good condition upon release.

### Fish Collection and Tagging

A detailed description of fish collection and tagging can be found in [20] and in [21]. Briefly, natural-origin steelhead smolts were collected at a weir across Big Beef Creek (2006–2010; N = 157), and at rotary screw traps in the South Fork Skokomish River (2006–2010; N = 98; hereafter “Skokomish River”), Hamma Hamma River (2007; N = 6) and the Dewatto River (2006, 2007; N = 54; Fig. 1) during the outmigration periods (April – June). Hatchery-raised smolts were removed at the eyed egg stage of development from wild steelhead redds in 2005 from the Hamma Hamma River, in 2007 from the Duckabush River and in 2007 and 2008 from the Skokomish River and reared to smolt stage at the Lilliwaup Hatchery (Hamma Hamma and Duckabush populations) and the McKernan Hatchery (Skokomish population). Hatchery smolt groups were released back into their river of origin (Hamma Hamma: N = 80; Skokomish: N = 154; Duckabush: N = 30). In total, 582 steelhead smolts were collected, tagged, and tracked using acoustic telemetry.

Wild smolts were tagged at the smolt collection locations on Big Beef Creek and the Skokomish River. Skokomish hatchery smolts were transported to the smolt trapping location on the Skokomish River, tagged, held for at least 24 hours, and released. Wild smolts were also collected, held, tagged, and released 24 hours later. Duckabush hatchery smolts were tagged at the Lilliwaup Hatchery, held for 24–96 hours, transported to the Duckabush River, and released. In 2006, each smolt was implanted with a V9 VEMCO transmitter (V9-2L-R64K, 9 mm diameter×20 mm length, 3.4 g weight, VEMCO, Ltd., Halifax, Nova Scotia). From 2007–2010 each smolt was implanted with a V7 transmitter (V7-2L-R64K 7 mm diameter×17.5 mm length, 1.4 g weight, VEMCO, Ltd., Halifax, Nova Scotia). Both V9 and V7 transmitters emitted unique codes at random intervals within a range of 30–90 seconds at a 69 kHz frequency. For details of the surgical tagging protocol, see [20].

### Receiver Arrays

VEMCO VR2 and VR3 receivers were deployed throughout the Hood Canal and Puget Sound to detect tagged smolts as they migrated from freshwater to open ocean. The detection range of acoustic receivers varies with transmitter size. VEMCO receivers typically detect V9 transmitters located within a radius of 400–500 m (VEMCO, [22]), and V7 transmitters within a radius of 200–300 m (VEMCO; [25]).

Four main acoustic receiver arrays were deployed linearly across-channel to detect tagged smolts at critical points during seaward migration (Table 1; Fig. 1). Four VR-2 receivers in 2006 (average of 580 m spacing to detect V9 tags) and seven VR-2 receivers in 2007, 2008, and 2010 (average of 330 m spacing to detect V7 tags) were suspended at regular intervals across the HCB to document migration through the northern end of Hood Canal. In 2009, the east half of the Hood Canal Bridge was being replaced and was absent (May 1 to June 3, 2009) during the peak of steelhead smolt migration. Therefore, only four receivers (350 m average spacing) were suspended from the west half of the HCB, and two receivers were deployed 1 kilometer south of where the bridge is normally anchored (Fig. 1). All other detection arrays were deployed upon the seafloor, fastened to both an anchor and a float, so that the device remained at a fixed location but was vertically oriented approximately 1 meter off the seafloor. In 2007, a line of seven receivers (420 m average spacing) was deployed near the middle section of Hood Canal (hereafter MCL) to evaluate behavior in lower Hood Canal. An array of 13 VR-3 receivers (460 m spacing) was deployed across Admiralty Inlet (ADM) in 2008, 2009, and 2010 to detect smolts passing through northern Puget Sound. A final array of 31 VR-2 receivers (760 m average spacing) spanned the Strait of Juan de Fuca (JDF) at Pillar Point (2006–2010) to detect smolts migrating out to the open ocean (Table 1; Fig. 1).

**Table 1:** Characteristics of the mid-canal (MCL), Hood Canal Bridge (HCB), Admiralty Inlet (ADM), and Strait of Juan de Fuca (JDF) acoustic telemetry receiver arrays.

	MCL	HCB	ADM	JDF
Years deployed	2007	2006–2010	2008–2010	2006–2010
Number of receivers	7	4 or 7*	13	31
Average spacing (m)	420	330–580*	460	760
Average waterdepth (m)				
Deployment method	Anchor**	Suspended	Anchor**	Anchor**
Number of smoltsdetected	109	357	50	67

* Receiver spacing in 2006 (580 m) accommodated V9 tags with a larger detection radius than V7 tags, and the east half of the HCB was absent in 2009 so fewer receivers were suspended from the bridge.

** Anchored receivers were moored approximately 1 meter above the substrate.

Several single receivers were deployed throughout Hood Canal each year. Two receivers were deployed at the mouth of Big Beef Creek, the Dewatto River, the Hamma Hamma River and the Skokomish River, and one at the mouth of the Duckabush River, to detect smolts as they entered Hood Canal. Varying numbers of additional receivers were deployed each year in Hood Canal (14 in 2006, 10 in 2007, 40 in 2008, 12 in 2009 and 13 in 2010), either suspended from US Coast Guard navigational aids or anchored to the seafloor (Fig. 1).

### Mortality Determinations and Behavior

**Figure 2:**
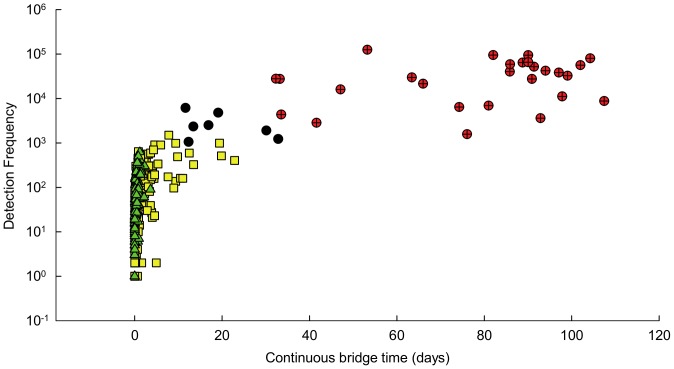
Scatterplot showing the distribution of continuous bridge time values and detection frequencies. Green triangles represent smolts categorized as ‘survivors’, yellow squares represent smolts categorized as ‘unknown’, a black dot represents a ‘possible mortality’, and a red dot with a black cross represents a smolt categorized as a ‘probable mortality’ (see text for criteria).

Smolts were categorized as “survivors” if they were detected at the ADM or JDF arrays, further along the migration route to the Pacific Ocean (Fig. 1). Smolts that were not detected at ADM or JDF (i.e., not certain to have survived) were categorized as either a probable mortality, possible mortality, or unknown based on two behavioral metrics: 1) total number of detections at the HCB, and 2) continuous time at the HCB. Continuous time at the HCB was calculated by summing all durations of continuous detections uninterrupted by an absence of greater than 24 hours or by detection on a non-HCB receiver. No survivor was detected at the HCB more than 620 times or for more than 3.6 continuous days (Fig. 2, Table 2); therefore, a smolt was conservatively categorized as a probable mortality if its tag was detected at the HCB more than 1,500 times and had a continuous HCB time of more than 30 days. Possible mortalities included smolts detected at least 1,000 times and with continuous bridge times of at least 10 days. Categorizing smolts into the possible category enabled us to be conservative in our mortality analysis while still demonstrating the abnormal behavior of several smolts at the HCB. Smolts categorized as ‘unknown’ had fewer than 1,000 detections at the HCB and continuous bridge times of less than 10 days, and were not detected at arrays beyond the HCB (Fig. 2).

**Table 2:** Mean (± SE) and (range) of behavioral parameters for steelhead smolts detected at the HCB in each of four categories that describe the inferred fates of individuals.

Category	N	Mean ± SE number of HCB detections	Mean continuous time ± SE at HCB (days)	Mean total time ± SE at HCB
Probable mortalities	27	38615±6303	77.8±4.5	79.1±4.3
		(1581–125141)	(32.3–107.4)	(32.3–107.4)
Possible mortalities	8	2706±641	20.0±2.9	30.6±5.2
		(1060–6140)	(11.6–32.8)	(12.3–45.5)
Survivors	94	88.0±12.1	0.5±0.1	2.6±0.7
		(1–620)	(0–3.6)	(0–42.8)
Unknown	228	132.9±13.6	1.5±0.2	4.6±0.7
		(1–1500)	(0–22.8)	(0–72.5)

Steelhead smolt behavior at each detection array (MCL, HCB, ADM and JDF) was quantified by calculating five additional behavioral metrics for each individual: (1) “time at array” was the time between the first and last detection at a particular receiver array; (2) “continuous time at array” (similar to continuous bridge time, described above) was calculated by summing all continuous periods of time an individual spent at a receiver line, with breaks in a continuum defined by a period of 24 hours with no detections or detection at a different (single or array) receiver; (3) “detection frequency” was simply the total number of times a smolt was detected at the receiver array; (4) “number of receivers” was the count of how many receivers a smolt was detected at the receiver array; and (5) “number of crossings” was the number of times a fish was detected sequentially at one end of a receiver array, then the other (i.e., back and forth behavior). Number of crossings was calculated only for the HCB and MCL, since the spacing and receiver number of only those two arrays were consistent enough to compare behavior (i.e, the HCB and MCL both consisted of 7 receivers and spanned a similar distance).

The five smolt behavior metrics were compared among receiver arrays, except that smolts detected at the HCB were divided into two categories: 1) “HCB mortality” to represent probable and possible mortalities and 2) “HCB” to represent known survivors and unknowns. The 2006 and 2009 data from smolts in the HCB and HCB mortality categories were excluded from the array comparisons because the receiver spacing or array geometry differed from the HCB spacing and geometry implemented in 2007, 2008 and 2010 (Table 1; sample sizes: MCL = 109, ADM = 50, JDF = 68, HCB mortality = 27, HCB = 193).

### Mortality Analysis

Exact tests of goodness-of-fit were calculated to test whether probable mortalities were randomly distributed among receivers or associated with the HCB. The frequencies of observed probable mortalities at the HCB and those at all other receivers were compared to the expected frequencies based on the ratio of HCB to non-HCB receivers. Separate tests were performed for each year, then observed and expected frequencies were combined for a pooled test. Since HCB receivers were located in the marine (as opposed to estuarine) environment and were suspended rather than anchored like most of the other deployed receivers, a second set of tests was carried out comparing the frequency of observed probable mortalities at the HCB to the observed probable mortalities only at other suspended marine receivers.

To determine whether probable mortalities were randomly distributed among populations, an intrinsic hypothesis G-test for goodness-of-fit was performed on the mortality frequency data. Observed probable mortalities were compared to expected frequencies based on the number of smolts from each population detected at the HCB. Populations with low expected frequencies were pooled to achieve acceptable values for the test (>5, [26]). Since prevalence of hybridization between steelhead and *Oncorhynchus clarki* cutthroat trout is high in Big Beef Creek (23.9% of phenotypic steelhead, [27]), and tagged steelhead in this study had been screened for diagnostic cutthroat alleles for a previous study [27], a G-test of independence was performed to determine whether hybrids from Big Beef Creek accounted for a larger proportion of mortalities than would be expected based on their occurrence at the HCB.

### Behavioral Analysis

General linear models (GLM) were constructed to test for differences in smolt behavior at each of the four detection arrays. Each of four models used detection array group (MCL, HCB, HCB mortality, ADM and JDF) as a fixed factor to partition variation in behavioral metric data (time at array, continuous time at array, detection frequency, and number of receivers). Behavioral metric data were all transformed using log or square-root calculations to improve normality and minimize differences in group variance. Tukey’s tests for multiple comparisons were used to test for significance between groups. Bridge crossing data were very skewed and could not be transformed to meet normality assumptions, so a Kruskal-Wallis non-parametric procedure was used to test for differences in back and forth behavior observed between the MCL, HCB and HCB mortality groups.

## Results

### Mortality

A total of 27 probable mortalities and 8 possible mortalities were documented at the HCB detection array (Table 3). One probable mortality was documented at a Big Beef Creek estuary receiver in 2008, but none of the 324 other non-HCB receivers recorded detections consistent with a probable or possible mortality throughout the study. Among the smolts detected at the HCB, 94 were categorized as survivors (later detected at ADM or JDF) and 228 were categorized as unknown (not detected at ADM or JDF).

**Table 3:** Determination of the fates of individual steelhead smolts implanted with acoustic telemetry transmitters (see text for criteria).

Year	Probable mortalities	Possible mortalities	Survivors	Unknown	Undetected at HCB
2006	4	3	34	41	25
2007	7	3	16	97	51
2008	4	1	19	38	47
2009	0	0	17	40	57
2010	12	1	8	12	45
TOTAL	27	8	94	228	225

Probabilities that probable mortalities were randomly distributed on all deployed receivers were very low each year (all p<0.001), and the probability was extremely low for all years combined (p = 5.97×10^−28^, Table 4). These low p-values indicate very strong evidence that mortalities observed in this study were associated with the Hood Canal Bridge, and that mortality at the HCB was greater than mortality rates at any other site we monitored. Even when expected ratios were calculated using only suspended receivers in the marine environment, probabilities of observing as many or more mortalities at the HCB were fairly low (though not significant at the 0.05 level in some years; Table 5).

**Table 4:** Results of exact tests for goodness-of-fit comparing the observed mortality ratios at the Hood Canal Bridge receiver array (HCB) to expected mortality ratios at all non-HCB receivers.

Year	Observed HCB mortalities	Observed non-HCB mortalities	Number of HCB receivers	Number of non-HCB receivers	P-value
2006	4	0	4	52	2.40×10^−5^
2007	7	0	7	62	1.07×10^−7^
2008	4	1	7	89	1.00×10^−3^
2009	0	0	4	62	NA
2010	12	0	7	60	1.60×10^−12^
POOLED	27	1	25	325	5.97×10^−28^

**Table 5:** Exact tests for goodness-of-fit comparing the observed mortality ratios to expected mortality ratios at non-anchored receivers.

Year	Probable HCB mortalities	Number of HCB receivers	Number of non-HCBmarine, suspended receivers	P-value
2006	4	4	3	0.034
2007	7	7	4	0.082
2008	4	7	7	0.063
2009	0	7	2	NA
2010	12	7	3	0.049
POOLED	27	25	17	5.17×10^−5^

No mortalities were detected on any of the non-HCB receivers.

Probable mortalities were non-randomly distributed among populations (G_adj_ = 26.22, df = 2, p<0.001). Twenty-two out of the 27 observed probable mortalities were smolts from Big Beef Creek (3 from the Hamma Hamma, and 1 from the Skokomish wild population), representing a much higher proportion than was expected based on the proportions detected at the HCB. However, hybrids were not more likely to be categorized as probable mortalities than were pure steelhead (G_adj_ = 0.10, df = 1, p = 0.748).

### Behavior

**Figure 3:**
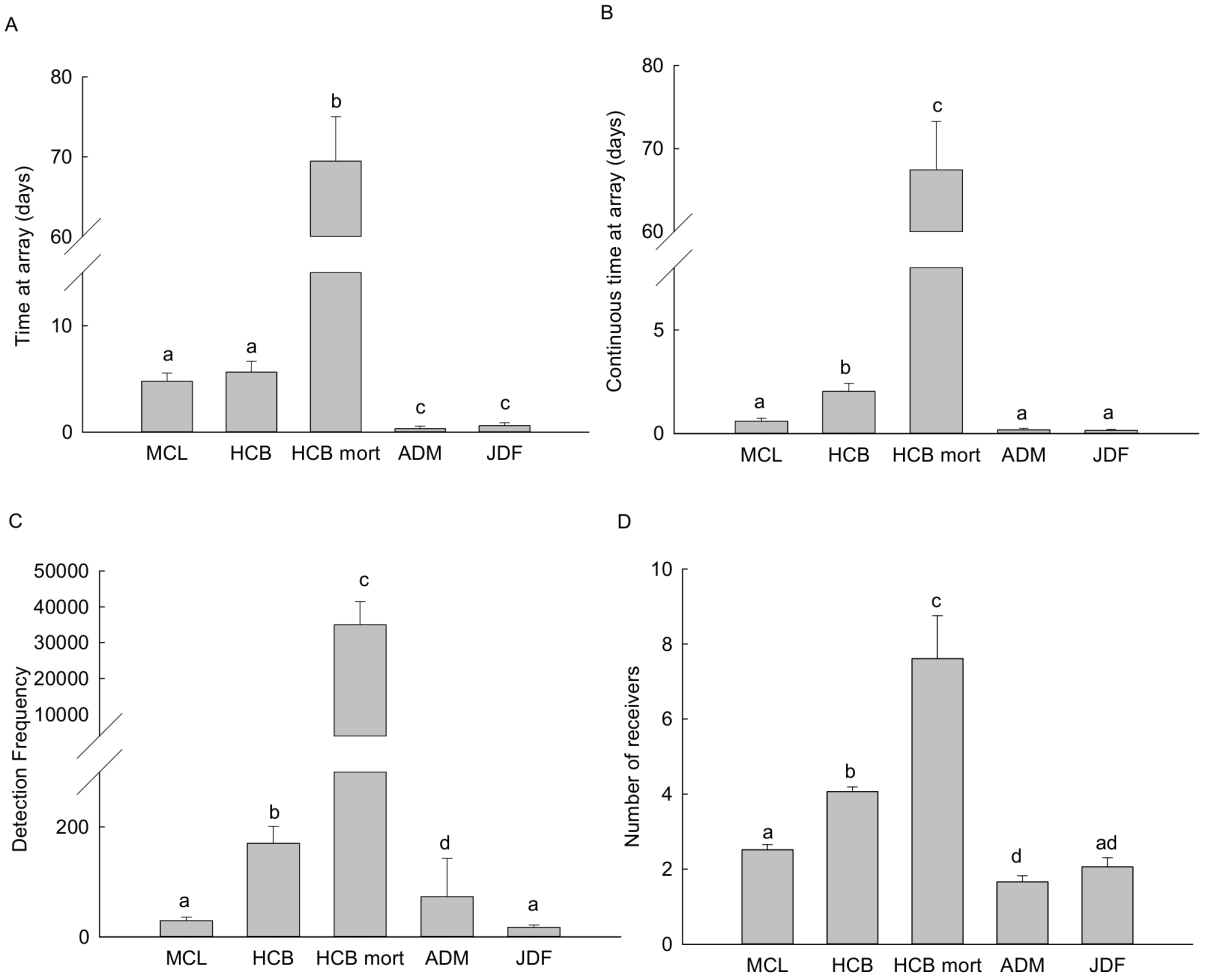
Steelhead smolt behavior at each receiver array (MCL, HCB, ADM, JDF). Behavior was measured by (a) total time at the array (± SE) from first to last detection, (b) continuous time at the array (± SE), which excludes any increments of time spent away from the array, (c) detection frequency (± SE) for each smolt at each array, and (d) total number of receivers (± SE) a smolt was recorded on. The HCB mort group is comprised of all smolts categorized as possible or probable mortalities. Different letters denote statistical significance.

**Figure 4:**
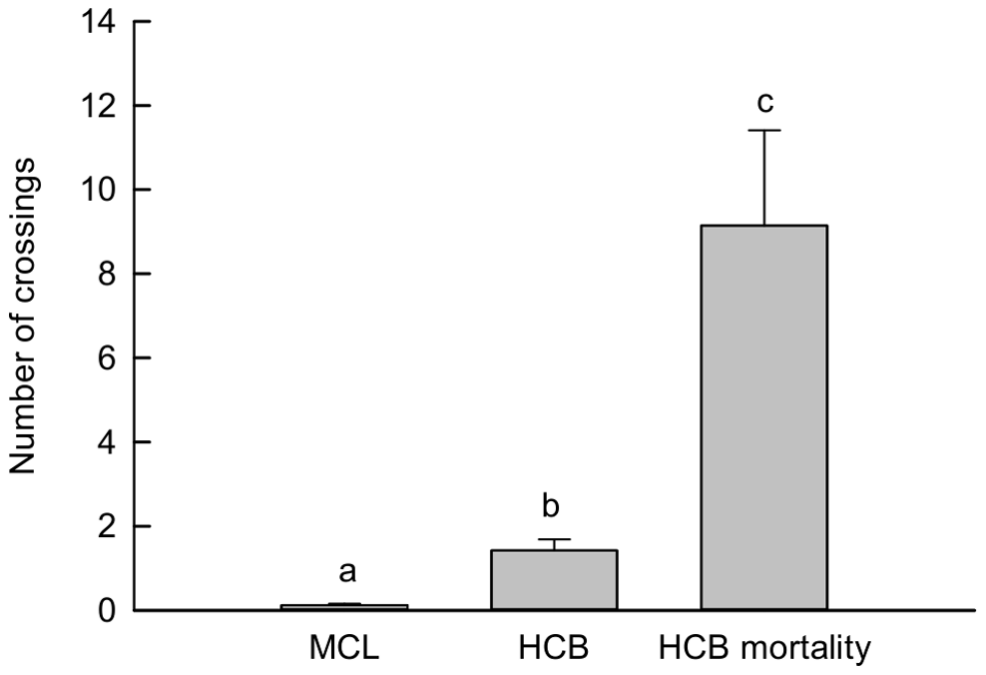
Counts of Hood Canal crossing events for steelhead smolt acoustic transmitters at the MCL and HCB receiver arrays. The HCB mortality group is comprised of all smolts categorized as possible or probable mortalities. Different letters denote statistical significance.

Detection array group was a significant factor in describing the variation in all five behavioral parameters (all p<0.001; Figs. 3A–D, Fig. 4). Time at array, continuous time at array, detection frequency and number of receivers were all significantly greater for smolts in the HCB mortality group than for HCB (non-mortality) smolts and smolts at all of the other arrays (all p>0.001, Fig. 3). Smolts spent similar amounts of time at the HCB and MCL arrays (p = 0.920), and spent significantly more time at these arrays than at either the ADM or JDF arrays (all p<0.001, Fig. 3A). In contrast, continuous time at array was significantly higher at the HCB than at the MCL, ADM and JDF arrays (all p<0.001, Fig. 3B). Detection frequencies were also greater at the HCB array than at the MCL, ADM and JDF arrays (all p<0.001, Fig. 3C), and detection frequencies were significantly greater at the ADM array than at the MCL and JDF arrays (p<0.001 and p = 0.029, respectively, Fig. 3C). Similarly, smolts were detected on significantly more receivers on the HCB array than on any of the other arrays (p<0.001, Fig. 3D). Smolts were detected on a similar number of different MCL and JDF array receivers (p = 0.006), and were detected on more MCL array receivers than ADM array receivers (p = 0.388; Fig. 3D). Smolts in the HCB mortality group crossed the HCB array more times than those in the HCB group (p<0.001), and smolts encountering the MCL array displayed significantly fewer crosses than smolts in the HCB group (p = 0.016, Fig. 4). The time between initiation and termination of crossing behavior ranged from 0.6–26.9 days (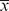 = 4.8).

## Discussion

Five years of acoustic telemetry data suggests that extra mortality of steelhead smolts migrating from Hood Canal to the Pacific Ocean occurred within several hundred meters of the Hood Canal Bridge. Over 5 years, 27 probable mortalities were recorded on 25 HCB telemetry receivers, while only one mortality was recorded on the remaining 325 receivers located throughout the Hood Canal, Admiralty Inlet, and the Strait of Juan de Fuca. Criteria for categorizing mortalities were fairly conservative given the behavior of known survivors, and evidence of extra mortality near the HCB is even stronger if possible mortalities (n = 8) are considered. Assuming telemetry-tagged smolts provided an unbiased representation of the larger Hood Canal steelhead smolt population, the HCB may directly or indirectly cause mortality of a minimum of 4.9% (2006) to 36.4% (2010) of the steelhead smolts migrating from Hood Canal (see Table 3). No probable or possible mortalities were observed during 2009 when half of the HCB was not present and an open migration corridor was available. Steelhead smolts detected at the Hood Canal Bridge exhibited significantly different behavioral patterns than did smolts detected at the other receiver arrays. Smolts encountering the HCB spent more continuous time, were detected more times, and were detected on more receivers than smolts at other arrays throughout the migration corridor. These behavioral parameters describe spatial and temporal aspects of smolt behavior, suggesting that the HCB is associated with both migration delay and increased migration distance for a portion of the population.

The time in between first and last detection (‘time at array’) was similar for smolts detected at the MCL and smolt survivors detected at the HCB, though significantly lower for smolts passing the ADM and JDF arrays. These results suggest that smolts in the Hood Canal migrate on a relatively tortuous path, meandering away then back toward the array more so than smolts migrating rapidly through Puget Sound and the Strait of Juan de Fuca. In contrast, smolts spent more continuous time at the HCB in relation to all other arrays, indicating that smolts were having difficulty migrating past that array in particular.

We hypothesize that behavioral anomalies of smolts detected on the HCB array provide indirect evidence that smolts encountering the Hood Canal Bridge experience a greater risk of predation than do smolts at the other arrays. Delays in migration and the number of array crossings suggest some difficulty navigating under or around the floating pontoons, and we assume that mortalities are the result of consumption by predators and defecation of the tags rather than other causes of mortality occurring within range of the HCB receivers. Melnychuk et al. [28] and La Croix et al. [29] came to the same conclusion regarding stationary tags encountered during their telemetry studies. Elevated predation rates have been documented at other major structures that inhibit movement of migrating salmon and cause unnaturally large aggregations. Sea lions at the Ballard Locks in Seattle, Washington [30] and at Bonneville Dam on the Columbia River [14] learned to target large groups of adult salmonids at migrational constrictions resulting from man-made partial fish barriers. Northern pikeminnow target portions of dam reservoirs on the Columbia River where juvenile salmonids are most densely distributed [13]. These examples describe an aggregative response of predators to prey, where predators increase local density in direct relation to increasing prey density [31]. While aggregative responses have been described for marine predators in natural (unaltered) habitats [32], [33], human-caused migration bottlenecks, such as those described above, may substantially reduce population viability (e.g., [30]).

Steelhead are known prey of *Phoca vitulina* (harbor seals) in marine waters along the US and Canadian Pacific coast [34]–[36]. Harbor seal predation on salmon and steelhead occurs opportunistically in accordance with abundance (aggregative response; [33], [34]. For example, peak abundance of Harbor seals in Netarts Bay, Oregon coincided with returning salmon runs at natural estuary constrictions [37]. Predation on salmon smolts is more difficult to observe than predation on adults due to differences in prey handling (underwater, versus above water for adults), but it has been documented [35]. Harbor seal populations in the Puget Sound region have increased exponentially since the 1970’s and are currently thought to be near carrying capacity [38]. Predation on salmon by Harbor seals and other pinnipeds is increasingly implicated as a possible factor limiting the recovery of imperiled Pacific salmon populations [39], [40]. Harbor seals haul out about 3 kilometers north of the HCB near the mouth of Port Gamble Bay [38], and are the most likely source of seals feeding near the HCB. More than 3 HCB crossings were never observed for any smolt in the group of survivors (median = 0), and more than 2 crossings were never observed at the MCL (median = 0). In contrast, the number of HCB crossings observed in the probable mortality group averaged 8 crossings (range = 0 - 39). Crossing behavior is thus unlikely to be performed multiple times by a steelhead smolt, and likely typical of the predator consuming smolts and transporting the transmitter back and forth along the HCB. Crossing back and forth along the HCB lasted an average of 4.8 days (range = 0.6–26 days), which is longer than the 6.5–29.7 h gut passage time reported for captive pinnipeds [41], but passage time of non-biological material through wild animals may be slower [42]. Piscivorous birds may also prey on steelhead smolts, though any telemetry tags carried by birds would not be detected continuously, and would not likely account for tags travelling back and forth across the HCB array because the acoustic pings cannot pass to the receivers through the air. Other potential non-piscine predators on steelhead smolts include *Lontra canadensis* (river otters), *Phocoena phocoena* (harbor porpoises), and *Zalophus californianus*. Unfortunately, we are unaware of any data on their presence at the HCB.

There are at least two plausible alternative explanations to the assumption that long periods of tag detections at one location result from predation and subsequent defecation. First, continuous detection of tagged smolts could reflect strong site fidelity. However, there are no known studies that have documented such behavior in salmonid smolts, and tags continuously recorded for more than 3.6 days were never detected on receivers farther along the migration route. The second explanation is that tags were being extruded from the body cavities of live smolts. If this were true, it would be extremely unlikely that virtually all tag extrusions occurred within the listening radius of the HCB receivers. A comprehensive laboratory study found that after 20 days post-implantation, steelhead smolts retained their VEMCO dummy tags at a high rate (97% for V7 tags and 95% for V9 tags) [43]. The mean time from release to last bridge detection in the present study was 16 days.

If the tagged group observed in this study did not represent the overall steelhead population due to tag effects, the impact of the HCB on steelhead populations may be overestimated here. A recent study conducted in the Columbia River estuary found acoustic-tagged salmon to be predated upon (presumably primarily by pinnipeds) at a higher rate than a comparable group of salmon with sham acoustic tags, indicating that predators were able to hear and detect the location of active-tagged fish [42]. However, this is the first study to report increased predation on acoustic tagged fish, and no further evidence exists supporting the same mechanism in juvenile salmon. Another study measuring smolt to adult survival of Columbia River Chinook salmon smolts found similar survival rates for one pair of acoustic- and PIT-tagged release groups and lower survival in the acoustic-tagged group relative to the PIT-tagged group for another population [44]. Additional evaluation must take place before we can determine whether increased predation on tagged fish presents an issue in this study.

Behavioral changes observed at the HCB could be induced by the physical structure of the bridge pontoons or by environmental factors altered by the structure, such as light level or surface flow patterns. If smolts are migrating at depths greater than 3.6 m (depth of bridge pontoons), they may not be affected at all by the HCB. At shallower depths, the physical barrier imposed by the pontoons may cause fish to be disoriented, block passage, and cause them to find an alternate route under or around the pontoons. Purse seine studies have shown that juvenile salmonids are generally found in the upper 12 m of the water column while migrating through the Columbia River estuary [24], but no finer scale information is available for smolts in marine waters. Steelhead adults travel in the upper 1.6 meters of marine waters [45] and steelhead smolts in freshwater are similarly surface-oriented (2.0 - 2.3 m; [46]). It is likely that the population displays variation in migration depth, so that some fish may be strongly affected by the bridge pontoons while others may migrate through the area undeterred.

The bridge may function to both attract smolts to the shade provided by the HCB while simultaneously inhibiting passage by disrupting Hood Canal currents. It is well established that fish utilize structures of many types to mitigate risk of predation [47], [48]. Steelhead smolts may be selecting the HCB environment and volitionally spending more time within the shaded habitat than they normally would along the otherwise open migration route. Disruption of prevailing currents in the Hood Canal may also impede normal steelhead migratory behavior. LaCroix et al. [29] recorded Atlantic salmon smolts predominantly using tidal currents to exit estuaries and bays along the North Atlantic coast. Currents may be a dominant influence on juvenile salmonid movement in coastal waters [49], and surface currents are certainly disrupted to some extent by the HCB pontoons. Current changes may send smolts off-course or induce the milling behavior observed at other physical migration barriers (e.g., [12]). Noise or vibration of vehicles travelling across the bridge deck, differences in prey density associated with the bridge structure, or altered chemical signature of the surrounding water due to vehicle emissions, may all contribute to the behavioral changes displayed by steelhead smolts at the HCB.

In summary, more steelhead smolt mortality events occurred within the vicinity of the Hood Canal Bridge than at any other site we monitored from 2006 through 2010. Smolts passing by the HCB receiver array behaved differently than those migrating past similarly spaced receiver arrays inside the Hood Canal, in Puget Sound, and in the Strait of Juan de Fuca. Behavioral changes could be a result of one or several interacting physical, ecological or environmental factors altered by the bridge structure. Mortalities are likely caused by predation by a marine mammal, inferred from movement patterns recorded on HCB receivers that would be atypical of surviving steelhead smolts or tags consumed by avian predators. This study identifies an area of particular concern for steelhead smolts migrating out of Hood Canal, and perhaps explains part of the reason for low early marine survival rates [20], [21]. Increasing understanding of the interactions between habitat modification, prey migration, and predator responses to aggregation may support management of similar migratory bottlenecks, and informs future decisions about development and increasing human infrastructure in aquatic environments.
